# Recent Paramedic Graduates’ Chronic Stress Adds Intentions to Leave the Profession: A Pilot Study Utilizing a Web-Based Survey

**DOI:** 10.1177/00469580231210706

**Published:** 2023-11-28

**Authors:** Anniina Herttuainen, Hilla Nordquist

**Affiliations:** 1 The Wellbeing Services County of South Savo, Mikkeli, Finland; 2 South-Eastern Finland University of Applied Sciences, Kotka, Finland

**Keywords:** ambulances, emergency medical services (EMS), paramedics, occupational stress, personnel turnover

## Abstract

Paramedics have mentally and physically demanding jobs, and chronic stress is not uncommon. Recently graduated paramedics, in particular, have been identified as needing support in their early careers. This pilot study examined chronic stress experienced by recent graduate paramedics and their intentions to leave the paramedic profession. Finnish paramedics encompass qualifications to work in various nursing sectors. This pilot study was a cross-sectional survey study among Finnish paramedics who graduated less than 3 years ago and who were currently working in prehospital EMS (n = 152). They evaluated chronic organizational and operational stressors on the Emergency Medical Services Chronic Stress Questionnaire with 20 statements. Two structured questions related to the intention to leave the paramedic profession. Three summary scales were formed. The differences in stress by the intention to leave were reported descriptively and the differences were tested with Mann-Whitney *U* test. The influence of potential predictors of the intention to leave prehospital EMS work or the nursing sector completely were explored with a forward stepwise logistic regression model. Those who intended to leave prehospital EMS work (25%, n = 35/152) or to leave the nursing sector completely (33%, n = 50/152) experienced higher levels of stress than those without such intentions. Stress related to organizational inequity and leadership challenges was the strongest and stress related to social, health, and personal impacts was the second strongest predictor of the intention to leave. Reducing chronic stress might be important in terms of paramedic retention. Several further study needs are addressed.

**What do we already know about this topic?**
Intentions to leave the nursing profession are common.
**How does your research contribute to the field?**
This pilot study is the first study examining chronic stress experienced by recent graduate paramedics and their intentions to leave the paramedic profession.
**What are your research’s implications toward theory, practice, or policy?**
The results of this pilot study raise several further study needs regarding efficiency demands and unique stressors of prehospital EMS work and leadership needs of newly graduated paramedics.

## Introduction

Paramedics work on call outside the hospital in varied environments, and their work is physically and mentally demanding.^1-3^ Paramedics’ experiences of stress have been widely studied.^3-10^ Chronic stressors are prolonged, threatening, or challenging conditions that interfere with daily life and continue for long periods of time, for at least a month.^11^ Donnelly et al divide chronic stress affecting paramedics into organizational and operational stress.^12^

Organizational stress manifests as conflicts with the employer, lack of support from the supervisor and colleagues,^3,7,13^ poor daily management, repetitive paperwork, and poorly managed organization of resources.^3,10^ Operational stress is caused by the conditions and uniqueness of the prehospital emergency medical service (EMS) work itself,^4,13^ including shift work with long work hours, little recovery time^3,10^ and sleep distruptions,^14^ but also impacts one’s identity, work-life balance and other social connections, as well as mental and physical health and overall well-being.^3,7,14-20^

Intentions to leave the profession are common and have been extensively studied from the perspective of recently graduated and young nurses, both internationally^17,21^ and in Finland.^22-25^ Studies focusing on paramedics are lacking, however, previous evidence has shown that experiences of stress among nurses affect their intention to leave the profession.^22,26-28^

Some of the stressors paramedics face in their work are unique compared to other occupations but are necessary to recognize in order to understand and support paramedics’ work abilities. Their recognized support needs, especially at the beginning of their careers, and the necessity of retaining paramedics in the prehospital EMS work, require awareness. To highlight the phenomenon, this pilot study examined chronic stress experienced by recent graduate paramedics and their intentions to leave the paramedic profession.

## Methods

This pilot study is a cross-sectional survey study with a target group of recently graduated Finnish paramedics (ie, at the time of the study, paramedics who graduated less than 3 years ago and were currently working in prehospital EMS). The Checklist for Reporting Results of Internet E-Surveys (CHERRIES) was used in reporting the results.^29^

### Setting

Finland is a Nordic country with approximately 5.5 million inhabitants, 780 000 EMS missions per year^30^ and 3900 paramedics.^31^ In Finland, the wellbeing services counties (at the time of the study, hospital districts) have the responsibility for organizing EMS. The national legislation and regional service standards determine the scope of EMS, as well as the level of training required for paramedics and the response times necessary to reach the majority of the population in different areas.^32,33^

Finnish paramedics work mainly in pairs in the ground-based EMS in basic and advanced level units. These units have slightly different care responsibilities, but the same on-call duties, and they generally have similar work rhythms, meaning shift work that, depending on the workplace, is most commonly in 12 or 24-h shifts.

Registered nurses without a prehospital specialization (bachelor’s degree in nursing = 3.5 years/210 ECTS [European Credit Transfer and Accumulation System] training at a University of Applied Sciences [UAS]), practical nurses with a prehospital specialization (3 years of training in vocational school, comparable to emergency medical technicians) and also professionally trained firefighters can work in the basic level units. To work at the advanced level, the paramedic must have a bachelor’s degree in emergency care (4 years of training/240ECTS in UAS, more than a third of the training involves clinical practice in various healthcare settings, as well as simulation exercises), or a bachelor’s degree in nursing with completion of a 30 ECTS advanced-level prehospital specialization course (30 ECTS in UAS). The graduates from these bachelor’s degrees are legally authorized as registered nurses by the Finnish National Supervisory Authority for Welfare and Health.^34-36^ In advanced level units, the second crew member can be a practical nurse, firefighter or an advanced level paramedic. Generally, Finnish paramedic education programs encompass broader qualifications to work in various sectors of nursing, not just prehospital EMS.

### Data Collection

The inclusion criteria of this study were: (1) being a paramedic with a bachelor’s degree in emergency care, a practical nurse with a prehospital specialization, or a registered nurse degree with or without the additional specialization studies, (2) having graduated less than 3 years ago at the time of the study, (3) currently working in prehospital EMS, and (4) user of social media (Facebook) targeted to Finnish prehospital EMS professionals. The exclusion criterion was individuals who did not meet all the inclusion criteria. To determine the appropriate sample size for this pilot study, we first utilized the formula n = (*Z*^2^ × *p* × (1 – *p*))/*E*^2^. Here, n represents the sample size, *Z* is 1.96 (corresponding to a confidence level of 95%), *p* is 0.10 or 10% (the estimated proportion of the target population), and *E* is 0.05 or 5% (representing the margin of error). Based on these parameters, our calculation yielded n = (1.96^2^_ _× 0.1 × (1 – 0.1))/0.05^2^, which is approximately 138. Given that our population was finite with N = 3898,^24^ we further adjusted the sample size using the formula n^adj^ = n / (1 + (n – 1)/N). This resulted in n^adj^ = 138/(1 + (138 – 1)/3898), which approximates to 133 recently graduated paramedics.

The data was collected using the Emergency Medical Services Chronic Stress Questionnaire, which has been developed and validated in Canada.^12^ The respondents rated the chronic organizational and operational stress they experienced during the last 6 months in response to 10 statements relating to each type of stress on a 7-point Likert scale (1 = no stress at all, 4 = moderate stress, and 7 = a lot of stress).^12^ Two questions concerned the intention to leave the paramedic profession: “I am seriously considering leaving prehospital EMS work during the next 2 years (moving to other positions in the nursing sector)” and “I am seriously considering leaving the nursing sector completely during the next 2 years.” The answer options were “Yes,” “No,” and “Not sure.” Gender, age, and educational background were included as background questions. The questionnaire had a total of 25 questions on 3 pages (5 + 10 + 10).

Ethical approval for the study was received on November 10, 2021 from the ethical committee of South-Eastern Finland University of Applied Sciences. The data was collected with an open survey from November 16, 2021 to November 30, 2021 using the Webropol survey software. The research was announced in 4 Facebook groups that include Finnish prehospital EMS professionals. The announcement detailed the survey’s target group, information about the study, participation based on informed voluntary consent, the anonymity of the responses, and the possibility of withdrawing and a link to the study’s privacy statement. A total of 152 responses were received.

### Statistical Methods

The background information is described in terms of frequencies and percentages. Frequencies and percentages below 5 are marked as <5.

An exploratory factor analysis was carried out prior to calculating summary scales. The factor analysis was performed separably for the organizational stress questions (Kaiser-Meyer-Olkin 0.875, Bartlett’s Test *P* = < .001) and for the operational stress questions (Kaiser-Meyer-Olkin 0.874, Bartlett’s Test *P* = < .001) following the original analysis instructions to analyze the 2 sets of questions separately.^12^ There were no missing values. The organizational stress questions were loaded into 2 factors, and the operational stress questions were loaded into one factor (Table 1). The Cronbach’s alpha values were good: .831, .851, and .882, respectively.

**Table 1.** Results of the Explanatory Factor Analysis of Chronic Organizational and Operational Stressors.

Questions	Organizational stressors		Operational stressors
	Factor 1	Factor 2	Factor 1
Staff shortages	**0.842**		-
Lack of resources	**0.789**		-
Bureaucratic red tape	**0.755**		-
Lack of training on new equipment	**0.678**		-
Constant changes in policy/legislation	**0.589**		-
Feeling like different rules apply to different people (eg, favoritism)		**0.812**	-
Feeling like you always have to prove yourself to the organization		**0.790**	-
Leaders over-emphasize the negatives (eg, supervisor evaluations, public complaints)	0.306	**0.734**	-
Dealing with supervisors		**0.726**	-
Unequal sharing of work responsibilities	0.418	**0.662**	-
Fatigue	*-*	-	**0.814**
Friends/family feel the effects of the stigma associated with your job	*-*	-	**0.805**
Managing your social life outside of work	*-*	-	**0.776**
Feeling like you are always on the job	*-*	-	**0.756**
Risk of being injured on the job	*-*	-	**0.712**
Shift work	-	-	**0.702**
Lack of understanding from your friends and family about your work	*-*	-	**0.665**
Negative comments from the public	*-*	-	**0.664**
Eating healthy at work	*-*	-	**0.638**
Making friends outside of the job	*-*	-	**0.633**

Three summary scales were formed accordingly and returned to the original range of 1 to 7. For clarity, the summary scales were named “stress related to challenges in efficiency” (Organizational stress/Factor 1, Table 1) and “stress related to organizational inequity and leadership challenges” (Organizational stress/Factor 2, Table 1), and “stress related to social, health, and personal impacts” (Operational stress/Factor 1, Table 1). ChatGPT was used in developing the names.

The normal distribution of the stress scores according to the intention to leave the prehospital EMS work (Yes / No + Not sure) and the intention to leave the nursing sector completely (Yes / No + Not sure) were tested with a Shapiro-Wilk test. Among the former, stress related to challenges in efficiency and stress related to organizational inequity and leadership challenges were not normally distributed in the “Yes”-group (n = 35, *P* = .13, *P* = .22, respectively), and among the latter, stress related to social, health, and personal impacts was not normally distributed in “No + Not sure”-group (n = 117, *P* = .18). Thus, the differences in stress by the intention to leave were reported with median (Md) and interquartile range (IQR), and the differences between groups were tested with a non-parametric Mann-Whitney U test, with a significance level of *P* = < .05.

The associations between independent variables and the “Yes,” intentions to leave the prehospital EMS work or nursing sector completely were explored with a forward stepwise logistic regression model. Gender (reference group men + other), education (reference group practical nurses), age group (reference group oldest), stress related to challenges in efficiency, stress related to organizational inequity and leadership challenges, and stress related to social, health, and personal impacts (on a scale of 1–7) were potential predictors. In each step, the predictors were selected based on statistical significance. Odd ratios with 95% confidence intervals (CI) are reported.

All the analyses were performed with SPSS version 28.

## Results

A total of 152 recently graduated paramedics responded to the survey, n = 99 (65%) of them were women, n = 52 (34%) were men, and <5 identified as “Other.” The largest age group was 25–29-year-olds (n = 68, 45%), followed by 30 to 34 (n = 28, 18%), <25 (n = 24, 16%), and ≥35 (n = 32, 21%) year-olds. One hundred thirteen participants (74%) were paramedics with a bachelor’s degree in emergency care, while 17 (11%) were registered nurses with a bachelor’s degree and a prehospital specialization. Other qualifications included being qualified as a practical nurse (9%) or as a registered nurse without the prehospital specialization (6%).

Thirty-five respondents (23%) had intentions to leave prehospital EMS work during the next 2 years, while 82 (54%) had not, and 35 (23%) were not sure. For the nursing sector completely, 50 (33%) had intentions to leave, 69 (45%) had not, and 33 (22%) were not sure.

There were clear statistically significant differences (*P* ≤ .003) in the amount of stress between those with intentions to leave and those with no intentions to leave or who were not sure about their intentions (Table 2). Those with intentions to leave had a higher level of stress in all studied groups. The highest score was found in stress related to organizational inequity and leadership challenges among those who had intentions to leave prehospital EMS work (median 5.40 (IQR 4.60, 6.20) on a scale of 1 to 7).

**Table 2.** Chronic Stress by the Intention to Leave the Paramedic Profession (n = 152).

	Stress related to challenges in efficiency	Stress related to organizational inequity and leadership challenges	Stress related to social, health, and personal impacts
	*P*-value Md (IQR)	*P*-value Md (IQR)	*P*-value Md (IQR)
Intention to leave prehospital EMS work	.003	<.001	<.001
Yes	4.40 (3.60, 5.80)	5.40 (4.60, 6.20)	4.60 (3.30, 5.60)
No/Not sure	3.80 (2.70, 4.80)	3.60 (2.40, 4.4)	3.20 (2.30, 4.10)
Intention to leave nursing sector completely	.002	<.001	<.001
Yes	4.5 (3.55, 5.80)	4.90 (3.55, 5.85)	4.00 (3.18, 5.00)
No/Not sure	3.70 (2.80, 4.80)	3.80 (2.40, 4.60)	3.10 (2.30, 4.10)

Stress related to organizational inequity and leadership challenges was the strongest predictor of intentions to leave prehospital EMS work (Table 3). All the stress types were more significant predictors than demographic variables. In the fourth and final logistic regression model, stress related to organizational inequity and leadership challenges (OR 4.19, 95% CI 2.28, 7.67) and stress related to social, health, and personal impacts (OR 2.22, 95% CI 1.36, 3.65) had a positive association with the intentions to leave prehospital EMS work. The results suggest that for every one-point increase in the stress score, the odds of the intentions to leave prehospital EMS work during the next 2 years were 4.19 and 2.22 times higher, respectively. However, stress related to challenges in efficiency (OR 0.54, 95% CI 0.32, 0.93) decreased the odds of the intentions to leave prehospital EMS work. In the final model, while adjusted for different types of stress, the odds of women intending to leave prehospital EMS work were 0.33 (95% CI 0.11, 0.97) times that of men and other genders. Education and age were not significant predictors of intention to leave prehospital EMS work.

**Table 3.** Results of Stepwise Logistic Regression Analyses, Predictors of the Intention to Leave Prehospital EMS Work and the Intention to Leave the Nursing Sector Completely, Separately (n = 152).

	OR	95% Confidence interval
*Intention to leave prehospital EMS work*		
Model 1.		
Stress related to organizational inequity and leadership challenges	3.29	2.13, 5.10
Model 2.		
Stress related to social, health, and personal impacts	1.62	1.09, 2.42
Stress related to organizational inequity and leadership challenges	2.93	1.85, 4.65
Model 3.		
Stress related to challenges in efficiency	0.59	0.36, 0.99
Stress related to social, health, and personal impacts	2.03	1.28, 3.21
Stress related to organizational inequity and leadership challenges	3.81	2.16, 6.73
Model 4.		
Being woman	0.33	0.11, 0.97
Stress related to challenges in efficiency	0.54	0.32, 0.93
Stress related to social, health, and personal impacts	2.22	1.36, 3.65
Stress related to organizational inequity and leadership challenges	4.19	2.28, 7.67
*Intention to leave nursing sector completely*		
Model 1.		
Stress related to organizational inequity and leadership challenges	1.71	1.31, 2.23
Model 2.		
Stress related to social, health, and personal impacts	1.41	1.03, 1.93
Stress related to organizational inequity and leadership challenges	1.50	1.12, 2.01

When considering the intention to leave the nursing sector completely, the only significant predictors in the second and final logistic regression model (Table 3) were stress related to organizational inequity and leadership challenges (OR 1.50, 95% CI 1.12, 2.01) and stress related to social, health, and personal impacts (OR 1.41, 95% CI 1.03, 1.93). Both increased the odds of intending to leave the nursing sector.

## Discussion

This pilot study examined chronic stress experienced by recently graduated paramedics and their intentions to leave the paramedic profession. Those who intended to leave prehospital EMS work or to leave the nursing sector completely experienced higher levels of stress. Stress related to organizational inequity and leadership challenges and stress related to social, health, and personal impacts had positive associations with the intention to leave prehospital EMS work, and stress related to challenges in efficiency, as well as being a woman had a negative association. In the case of having intentions to leave the nursing sector completely, associations were found for stress related to organizational inequity and leadership challenges and stress related to social, health, and personal impacts. Notably, more paramedics were considering leaving the nursing sector completely rather than changing from prehospital EMS work to other positions in the nursing sector during the next 2 years.

The results infer an association between chronic stress and the intention to leave the paramedic profession, but causality could not be demonstrated in this pilot study. Generally, survey studies of working conditions in Finland have reported that a considerable number of young nurses have considered leaving the profession.^25^ Overall, nurses under 35 years old are the most dissatisfied.^37^ According to these reports, for example, career development, salary, well-being at work, and staff resources do not meet expectations.^25,37^ In the current study, stress related to organizational inequity and leadership challenges was the most significant predictor of the intention to leave and could be interpreted as partly reflecting these types of issues. A recent review of the intentions of critical care nurses to leave the profession found that leadership challenges were connected to increased stress and equality-related workplace experiences were linked to no intentions to leave.^38^ In line with this, a recent systematic review focusing on EMS found that supervisor support is a main predictor of job satisfaction and is connected to professional commitment.^39^ In addition, a recent survey study among emergency medical technicians found that supportive leadership reduced the odds of the intentions to leave.^40^ Moreover, a recent Finnish study among paramedics found that unsupportive management and feelings of inequality were connected to the experience of stress,^2^ but the study did not focus on intentions to leave. However, some of this sort of stress may reflect the overall uncertainty of a new professional. Hörberg et al found that newly graduated paramedics desire defined expectations from the organization and managers,^41^ and Ericsson et al found that performance expectations are one of the demands of the paramedic profession.^2^ Future studies should investigate in more detail what kind of leadership recently graduated paramedics need, and efforts should be made to enhance the alignment between actual circumstances and the needs. Attention should also be paid to the implementation of equality within the organization, and examine, for example, whether the phenomenon is a matter of attitudes or a lack of competence.

This pilot study’s target group consisted of recently graduated paramedics, and overall, the number of chronic stress experiences is worrying. According to previous studies in different settings, the chronic stress of paramedics and nurses is often caused by exhaustion, fatigue, insufficient recovery,^5,8,10,22,23^ and shift work.^10,21,23^ Other studies in the nursing sector have linked chronic stress emerging from these types of issues to recently graduated nurses’ intentions to leave the profession.^22,23,25^ Overall, the role of a paramedic is inherently challenging.^1-3,42^ Therefore, it is essential to maintain and nurture a balance between work-related demands and the social, health, and personal resources of new professionals. This balance not only concerns professional obligations but also personal life. Ericsson et al have recognized that achieving a good balance between work and personal life is a valuable resource for paramedics.^2^ Pursuing this should begin during paramedic training, where it’s crucial to equip students with strategies to face and cope with chronic stress. The paramedic curricula should encompass, for example, familiarization, mentoring, feedback,^21-25,41,43^ and emotional working skills.^44^ This would ensure that paramedics are better prepared to handle stressful events efficiently^45^ and recognize and address potential feelings of identity disruption.^15,16^ In addition, understanding the significance of support from colleagues,^7,23^ family, and friends,^5^ and the value of preventive routines, such as short breaks during shifts, feedback sessions after missions,^4^ and sufficient sleep^14^ in alleviating stress. Moreover, paramedics also need organizational support and informal defusing sessions.^9,10,46^ However, considering the unique nature of paramedic work, the phenomenon would benefit from further studies to form a comprehensive picture and to plan and execute effective, timely preventive measures and interventions.

The work of paramedics inherently requires maintaining and utilization of various competencies in constantly changing working conditions,^47-49^ and those with less work experience may feel pressure in mastering all the required content.^41,50^ In addition to this, the job involves a continuous time pressure, and the work pace has also intensified.^2,39,42^ As a continuum, severe forms of work disability, for example burnout, are not uncommon among paramedics.^19,20^ The challenging pandemic situation increased workloads and mental burden in the nursing sector,^51,52^ and could have increased the experiences of stress also in this study, which was conducted during the pandemic. However, according to the results, the stress related to the challenges in efficiency was negatively associated with intentions to leave prehospital EMS work. One possible explanation for the result is that the resources and work schedule in EMS work are still considered different and more pleasant than in other jobs in the nursing sector. Nevertheless, these kinds of associations require further studies among paramedics. It is still important to notice that rather than transitioning from prehospital EMS work to other roles within the nursing sector, more paramedics had intentions of leaving the nursing sector completely. This both reflects deep dissatisfaction toward the field in general^25^ but implies that the chosen specialty is still considered a better fit, although all the Finnish paramedics are qualified to work in a wide range of nursing roles. Previous studies have emphasized that paramedics possess a distinctive professional identity differentiating them from other healthcare professionals.^3,15,16,53^ There is evidence that, for example, the desire to help, the opportunity to save lives, passion for the field, curiosity, and diversity of tasks are keeping the paramedics in their profession.^3^ Generally, work should not induce such chronic stress that leads one to question their choice of profession that required years of education. Based on the results of this pilot study, the subject demands and deserves more extensive further research. The outcomes of such studies should be utilized in shaping the work to better preserve the well-being of paramedics.

### Methodological Considerations

Paramedics are a small professional group in the Finnish social and health sector.^31^ It can be assumed that in 2021, individual EMS organizations had significantly more experienced, long-term employees than new graduates. In our pilot study, we approximated that 10% of paramedics working in prehospital emergency care in 2021 were recent graduates who could be reached via social media targeted at Finnish prehospital EMS professionals. Yet, comprehensive public data detailing graduation years, professional experience, or other demographics of paramedics in this sector is lacking. As such, precise numbers of paramedics who met the inclusion criteria of this study remain elusive. With the estimate of 10% and sample size calculation result of 133, the 152 survey respondents were a reasonable sample size for this pilot study.

In this pilot study, social media was used to inform potential participants about the study due to the challenging accessibility of the newly graduated paramedics. It was estimated that it would be possible to reach many more recently graduated paramedics from a wider area through social media than, for example, through the internal processes of numerous emergency care service organizations.^54^ However, using social media in the collection of data can cause limitations. First, it was possible to reach only those using the selected social media groups. Second, inclusion in the target group was based on the respondents’ self-assessment. Due to the survey’s anonymity, researchers were unable to confirm participants’ eligibility. Third, more of those who had especially experienced stress or considered leaving the paramedic profession or who otherwise found the topic interesting might have responded than those without such interests. Consequently, the sample is a convenience sample. These pilot study limitations should be addressed in subsequent larger studies, aiming for a more precise sample size determination and a more randomized selection, for example, by conducting data collection at the organizational level.

However, the data collection had several strengths. The shortness of the survey and thus the faster response time, as well as the researcher’s reminder messages in the Facebook announcements, probably increased the number of respondents.^55^ In the literature, the connection between rewarding respondents to a better response rate for Internet-based surveys has been identified,^55,56^ but in this pilot study, the respondents were not offered rewards or organized raffles, as maintaining total anonymity was seen as more valuable. Conversely, this choice also supports the fact that the respondents were interested in the survey because they belonged to the target group.

This pilot study utilized a previously developed and validated questionnaire.^12^ Although the questionnaire was developed in Canada, the stressors are universal among EMS professionals. The survey was anonymous, and IP (Internet Protocol) addresses or repeat visits by the same individual to the survey page were not tracked. The survey was opened to the cover letter stage 644 times, and answering was initiated 194 times. Only completed questionnaires were included in the data, as the questions were mandatory, so all respondents answered each question before they were able to submit. The respondents had an opportunity to review and change their answers before submitting. In this pilot study, the results were analyzed slightly differently from what the original questionnaire instructions suggested. This was done to make the results as informative as possible for the design of future more detailed studies. All the questions from the original questionnaire were included in the summary scales formed in this pilot study.

The results of this pilot study are encouraging that this topic would be feasible for an in-depth examination with random samples of paramedics or qualitative research methods.

## Conclusions

In this pilot study, higher levels of chronic stress and intentions to leave the paramedic profession were associated among recently graduated paramedics. Especially stress related to organizational inequity, leadership challenges, and social, health, and personal impacts were found to be predictors of intentions to leave the profession.

The results of this pilot study raise several further study needs in keeping trained paramedics in their work. Attention should be given to the leadership needs of newly graduated paramedics and evaluate fairness within their organizations and determine whether any issues are due to attitudes or lack of skills, which could be improved via training. In addition, further research is needed on the unique stressors of prehospital EMS work to better understand these challenges and guide the development of effective preventive measures. Moreover, there might be a need for developing comprehensive support systems and strategies tailored specifically for newly graduated paramedics to better manage the chronic stress associated with their work. Future studies could also explore how efficiency demands influence paramedics’ intentions to leave the profession and whether these experiences differ compared to other sectors in nursing.
